# Draft Genome Sequence of *Lactobacillus animalis* 381-IL-28

**DOI:** 10.1128/genomeA.00478-14

**Published:** 2014-05-29

**Authors:** Joseph M. Sturino, Mahitha Rajendran, Eric Altermann

**Affiliations:** a Nutrition and Food Science Department, Texas A&M University, College Station, Texas, USA; b Intercollegiate Genetics Program, Texas A&M University, College Station, Texas, USA; c AgResearch Ltd., Palmerston North, New Zealand; d Riddet Institute, Massey University, Palmerston North, New Zealand

## Abstract

*Lactobacillus animalis* 381-IL-28 is an integral component of a multistrain commercial culture with food biopreservative and pathogen biocontrol functionality. A draft sequence of the *L. animalis* 381-IL-28 genome is described in this paper.

## GENOME ANNOUNCEMENT

*Lactobacillus animalis* 381-IL-28 is a component of a commercial biocontrol culture. Similar to probiotics (1), biocontrol cultures are living microorganisms that, when applied in adequate amounts, extend the safe storage life of beverages, foods, or feeds without changing their organoleptic properties (2). Some *L. animalis* strains are generally recognized as safe for the biocontrol of *Campylobacter*, *Escherichia coli* O157:H7, and *Salmonella* organisms in meat and poultry products (3–5) and on fresh-cut spinach (6). The *L. animalis* 381-IL-28 genome was sequenced to determine the genetic basis of its antimicrobial characteristics.

In brief, *L. animalis* 381-IL-28 was cultivated in Menon-Sturino (MS) broth supplemented with 100 mM d-glucose (7), and the genomic DNA was isolated by alkaline lysis (MasterPure Gram-positive DNA purification kit; Epicentre, Madison, WI), ethanol precipitation (8), and solid phase extraction (DNeasy blood purification kit; Qiagen, Valencia, CA). Genomic DNAs were sequenced using two chemistries. A paired-end library was prepared and sequenced using a Genome Analyzer IIx (Illumina, San Diego, CA) at the Genomics and Bioinformatics Center (College Station, TX). Genomic DNA was also primed for shotgun sequencing (Ion Xpress template kit; Ion Torrent, Grand Island, NY), and the library was sequenced by Epoch Life Science, Inc. (Sugar Land, TX) using a Personal Genome Machine (Ion Torrent).

The reads were randomly downsampled using the computational genomics (CG) pipeline (9) to an average 100-fold coverage and assembled *de novo* using Velvet (10) and VelvetOptimiser (11). The assembly was manually validated with AMOS and Hawkeye (12), and read coverage was assessed using SMALT (http://www.sanger.ac.uk/resources/software/smalt/) and SAMtools (13). The final assembly comprised 12 scaffolds (32 contigs) and 68 contigs (1,858,297 nucleotides [nt]). Twenty-seven contigs >10,000 nt covered 97% of the draft genome. Most contigs (93%) were within two standard deviations of the average coverage (110-fold), while the minimum coverage was 39-fold.

The size (1.86 Mb) and G+C content (41.1%) of the *L. animalis* 381-IL-28 draft genome were compared to those of other previously sequenced members of the *Lactobacillus salivarius* group, including *L. animalis* KCTC 3501 (1.88 Mb, 41.1% G+C) (16) and *L. salivarius* UCC118 (2.13 Mb, 33% G+C) (17). A functional genome distribution (FGD) analysis (14) was carried out and genome synteny visualized using ACT (15). Gene synteny differed among *L. animalis* 381-IL-28, *L. salivarius* UCC118, and other *L. salivarius* strains. Furthermore, 381-IL-28 harbors 549 strain-specific genes not found in *L. salivarius* UCC118 (e-value cutoff, 1e^−10^). In contrast, an FGD comparison between *L. animalis* strains 381-IL-28 and KCTC 3501 showed a high degree of gene synteny within the contigs. An ORFeome comparison highlighted 179 *L. animalis* 381-IL-28-specific genes (e-value cutoff, e^−100^), including an integrated prophage (open reading frames [ORFs] 822 to 877), transposase elements, and a clustered regularly interspaced short palindromic repeat (CRISPR) system (ORFs 1279 to 1288).

Protein-coding domain sequences were predicted and the draft genome was annotated using GAMOLA version 2 (18). Three l-lactate dehydrogenases (EC 1.1.1.27; ORF_1417, ORF_1456, ORF_1601) and one acetaldehyde dehydrogenase (EC 1.2.1.10; ORF_1385) were among the 1,844 protein-coding genes that were predicted.

### Nucleotide sequence accession numbers.

This whole-genome sequencing project was deposited at DDBJ/EMBL/GenBank under the accession no. JMHU00000000. The version described in this manuscript is the first version, JMHU01000000.
